# Perioperative albumin *versus* other fluids to prevent cardiac surgery-associated kidney injury: a systematic review and meta-analysis of randomized trials

**DOI:** 10.62675/2965-2774.20260353

**Published:** 2026-07-01

**Authors:** Phoebe R Darlison, Yahya Shehabi, Humphrey G. M. Walker, Ary Serpa, David C. Motorniak, Adrian Pakavakis, Mayurathan Balachandran, Geoffrey J. Wigmore, Rinaldo Bellomo, Alastair J. W. Brown

**Affiliations:** 1 Department of Anaesthesia, Perioperative Medicine, and Pain Medicine Peter MacCallum Cancer Centre Melbourne Victoria Australia Department of Anaesthesia, Perioperative Medicine, and Pain Medicine, Peter MacCallum Cancer Centre - Melbourne, Victoria, Australia.; 2 Department of Critical Care Medicine St Vincent’s Hospital Melbourne Fitzroy Victoria Australia Department of Critical Care Medicine, St Vincent’s Hospital Melbourne - Fitzroy, Victoria, Australia.; 3 Sir Peter MacCallum Department of Oncology The University of Melbourne Melbourne Victoria Australia Sir Peter MacCallum Department of Oncology, The University of Melbourne - Melbourne, Victoria, Australia; 4 School of Clinical Sciences at Monash Health Monash University Clayton Victoria Australia School of Clinical Sciences at Monash Health, Monash University - Clayton, Victoria, Australia.; 5 Department of Intensive Care Monash Medical Centre Clayton Victoria Australia Department of Intensive Care, Monash Medical Centre - Clayton, Victoria, Australia.; 6 Department of Intensive Care The Victorian Heart Hospital Clayton Victoria Australia Department of Intensive Care, The Victorian Heart Hospital - Clayton, Victoria, Australia.; 7 Prince of Wales Clinical School of Medicine University of New South Wales Randwick New South Wales Australia Prince of Wales Clinical School of Medicine, University of New South Wales - Randwick, New South Wales, Australia.; 8 Department of Critical Care University of Melbourne Melbourne Victoria Australia Department of Critical Care, University of Melbourne - Melbourne, Victoria, Australia.; 9 Australia and New Zealand Intensive Care Research Centre Monash University Clayton Victoria Australia Australia and New Zealand Intensive Care Research Centre, Monash University - Clayton, Victoria, Australia.; 10 Intensive Care Unit Austin Health Heidelberg Victoria Australia Intensive Care Unit, Austin Health - Heidelberg, Victoria, Australia.; 11 Department of Critical Care Medicine Hospital Israelita Albert Einstein São Paulo SP Brazil Department of Critical Care Medicine, Hospital Israelita Albert Einstein - São Paulo (SP), Brazil.; 12 Faculty of Medicine, Nursing and Health Sciences Monash University Clayton Victoria Australia Faculty of Medicine, Nursing and Health Sciences, Monash University - Clayton, Victoria, Australia.

**Keywords:** Albumin, Acute kidney injury, Cardiac surgery, Fluid therapy

## Abstract

**Objective:**

Cardiac surgery-associated acute kidney injury is a common and serious complication of cardiac surgery. Albumin solution is a commonly administered fluid in cardiac surgery patients; the role of albumin in preventing cardiac surgery-associated acute kidney injury is unclear. The objective of this systematic review and meta-analysis was to evaluate the impact of perioperative albumin compared with other fluid regimens on the risk of acute kidney injury in cardiac surgical patients undergoing cardiopulmonary bypass.

**Methods:**

A systematic search was performed of MEDLINE^®^, Embase, CINAHL, and Cochrane Central Register of Controlled Trials databases, and the Australian New Zealand Clinical Trials Registry, ClinicalTrials.gov, World Health Organization International Clinical Trials Registry Platform, and ISRCTN registries. Randomized trials of adult patients undergoing on-bypass cardiac surgery comparing albumin-containing solutions with any other fluid regimen given perioperatively were included. Trials comparing fluids used only for bypass priming were excluded. Data extraction, risk of bias, and certainty of evidence were assessed in duplicate by independent reviewers. A Bayesian framework was the primary statistical approach, with a secondary frequentist approach. The primary outcome was perioperative acute kidney injury, defined as the period from surgery until hospital discharge. Secondary outcomes were all-cause mortality at longest follow-up, intensive care unit length of stay, hospital length of stay, proportion of patients requiring renal replacement therapy postoperatively, duration of mechanical ventilation postoperatively, and duration of vasopressor support postoperatively.

**Results:**

Fourteen randomized trials, including 3,304 adults, were included in the analysis. Seven trials contributed data to the primary outcome. Four trials had an overall low risk of bias across all domains and outcomes. The pooled estimated risk ratio for acute kidney injury with albumin solutions was 1.09 (95% credible interval 0.86 - 1.34, tau = 0.12; I2 = 31.5%), with a 18.3% posterior probability of reduced acute kidney injury. There were no significant subgroup effects or differences in secondary outcomes.

**Conclusion:**

Among patients undergoing on-bypass cardiac surgery, the use of albumin solutions is unlikely to reduce the risk of acute kidney injury. Other interventions need to be considered for this condition.

## INTRODUCTION

Cardiac surgery-associated acute kidney injury (CSA-AKI) is a common complication affecting 20-40% of cardiac surgery patients.^(1,2)^The pathophysiology of CSA-AKI is complex, and risk factors are multifactorial.^(1,2)^Moreover, CSA-AKI is associated with adverse patient-centred outcomes, including long-term renal dysfunction and increased mortality.^(1,3,4)^ Presently, therapies for CSA-AKI are limited to mitigation of risk factors and supportive care.^(1,2,5)^

Albumin solutions are commonly administered during and after cardiac surgery. One multicentre Canadian audit found that cardiac surgery was among the most common specialties in which albumin was prescribed.^(6)^ Similarly, in the United States, one study found albumin was most commonly used in cardiovascular intensive care units (ICUs) compared with any other ICU.^(7)^Current literature suggests some variability in clinical practice across different healthcare systems globally. One post-hoc analysis of a multicentre Canadian trial of cardiac surgery patients with postoperative bleeding found 71% of all participants received albumin at least once postoperatively.^(8)^ This is consistent with practice surveys from Canada and the United States, which found albumin was used by 62% of Canadian respondents,^(9)^and in the United States, albumin was chosen as first-line fluid therapy during cardiopulmonary bypass by 37% to 56% of respondents.^(10)^ In the Australian and New Zealand context, a multicentre prospective observational study found 4% albumin solution was the second most common choice for fluid boluses post-cardiac surgery.^(11)^ Conversely, in a European survey of cardiac surgical anaesthetists, albumin use was less common than described in other continents, with 11% of respondents using albumin for postoperative volume expansion.^(12)^

Albumin solutions have been proposed to improve outcomes by optimizing fluid balance, preserving the endothelial glycocalyx, and mitigating the activation of inflammatory pathways and oxidative stress.^(13-16)^Despite these potential benefits, previous systematic reviews have not supported the use of albumin to protect renal function in the cardiac surgery population.^(17-19)^The current International Collaboration for Transfusion Medicine Guidelines (ICTMG) recommend against the use of albumin to prevent acute kidney injury (AKI) in major surgery.^(20)^ However, the strength of the recommendation was limited by small, heterogeneous studies.

Since these recommendations, two randomized clinical trials have been conducted: HAS FLAIR II^(21)^ and ALBICS-AKI.^(22)^ Considering these recent large-scale studies, this meta-analysis sought to address the limitations described in previous reviews,^(19,20,23)^incorporating new data for over 1,000 participants, the inclusion of high-risk populations, and the novel inclusion of trials investigating the use of hyperoncotic albumin solutions.^(21,22)^

A distinction in methodology compared with previous reviews was the exclusion of trials evaluating off-bypass cardiac surgical populations in the current review. This was intended to focus on the impact of albumin fluid therapy in the context of cardiopulmonary bypass, an intervention with unique risk factors associated with CSA-AKI.^(1,2,5,24)^ Additionally, the statistical approach, with a primary Bayesian framework, enabled assessment of the probability of a treatment effect in the context of low event rates. In contrast, the secondary frequentist approach provided a comparative sensitivity analysis.

Consequently, this updated systematic review and Bayesian meta-analysis were conducted to evaluate the impact of perioperative albumin compared with other fluid regimens on the risk of acute kidney injury in cardiac surgical patients undergoing cardiopulmonary bypass.

## METHODS

A systematic review of randomized clinical trials was conducted according to a prespecified, published protocol. The review was prospectively registered with the International Prospective Register of Systematic Reviews (PROSPERO) (CRD42024580170) and was reported according to the Preferred Reporting Items for Systematic Review and Meta-Analyses (PRISMA) statement.^(25)^

### Eligibility

Randomized trials that recruited adult patients undergoing cardiac surgery on cardiopulmonary bypass and compared albumin-containing solutions with any other fluid regimen given intraoperatively or postoperatively were included. Trials comparing fluids used for bypass priming only were excluded. Studies with at least one of the prespecified primary or secondary outcome measures were included. Any dose or duration of intervention fluids was eligible. Albumin-containing solutions included any human albumin preparation, and eligible comparators were defined as any fluid regimen different from the intervention fluid. Studies with a subset of eligible participants were eligible if subgroup analysis data were available.

### Search strategy

A systematic search of MEDLINE^®^, Embase, Cumulative Index to Nursing and Allied Health Literature (CINAHL), and the Cochrane Central Register of Controlled Trials was performed on 23^rd^ August 2024. A search of the Australian New Zealand Clinical Trials Registry, ClinicalTrials.gov, the World Health Organization International Clinical Trials Registry Platforms, and the International Standard Randomized Controlled Trial Number (ISRCTN) registry was performed between 2^nd^ and 5^th^ September 2024. The search included results from the inception of each database with no restrictions on language, publication status, or date. Search terms were created by one reviewer in consultation with a research librarian and experts in critical care and fluid therapy.

The detailed search strategy for each database and registry is presented in Supplementary Material. A manual search of the bibliographic references of relevant trials and reviews was conducted to identify additional references. Trial authors were contacted on at least two occasions when necessary for further information.

### Study selection

Screening of all identified references was conducted in duplicate using Covidence systematic review software.^(26)^ Duplicate references and non-randomized trials were automatically excluded using Covidence software. A minimum of two reviewers independently screened all remaining records using the study title and abstract to identify potentially eligible studies. Full-text publications and study reports were retrieved, and at least two reviewers independently screened them for eligibility according to prespecified inclusion criteria. Disagreements in study selection were resolved by consensus.

### Data collection

Data extraction was performed using the Covidence data system.^(26)^ Data from each study were extracted in duplicate by two independent reviewers using a piloted data extraction template. Discrepancies in data extraction were resolved by consensus. Authors of included trials were contacted, where possible, to request missing aggregate-level data. Aggregate-level data were provided prior to the publication of one trial by the corresponding author.^(22)^ Missing data were not imputed.

### Risk of bias assessment

Risk of bias for each trial was assessed by two independent reviewers in duplicate using the Cochrane Risk of Bias tool for randomized trials version 2 (ROB2).^(27)^ Members of the review team did not assess studies in which they were investigators. The risk of bias was assessed for all outcomes of interest. Discrepancies were resolved through consensus among reviewers.

### Outcomes

The primary outcome was perioperative AKI, defined as the period from surgery until hospital discharge. Definitions used in the original trials were unified using the Kidney Disease Improving Global Outcomes (KDIGO) criteria, as detailed in the review protocol.^(5)^ Data were also collected for the following prespecified secondary outcomes: all-cause mortality at longest follow-up, proportion of patients requiring renal replacement therapy (RRT) postoperatively, duration of mechanical ventilation, duration of ICU admission, duration of hospital admission, and duration of vasopressor medication postoperatively.

### Data synthesis

A Bayesian framework was used as the primary statistical approach, with a frequentist approach as the secondary approach. A random-effect model was used in the analyses. Pooled estimates of effect sizes were presented as risk ratios (RRs) for binary outcomes, and mean differences (MD) for continuous outcomes. Continuous variables presented in formats not readily amenable to pooling were converted to means and standard deviations (SDs) as described elsewhere.^(28)^ Along with the pooled estimates of effect sizes, 95% credible intervals (95%CrI) for the Bayesian meta-analysis and 95% confidence intervals (95%CI) for the frequentist model were presented. The 95%CrI were calculated using the shortest-interval method, which, for unimodal posteriors, is equivalent to the highest posterior density region. For all analyses, Bayes factors were based on marginal likelihoods. Trials with zero events were included in the final model, and an effect estimate was calculated accordingly.

For the frequentist analysis, a random-effect model using DerSimonian-Laird estimates of the between-study variance was used. As a further sensitivity analysis, frequentist analyses were performed using a random-effects meta-analysis with weights calculated according to the Mantel-Haenszel method.

**Figure 1:**
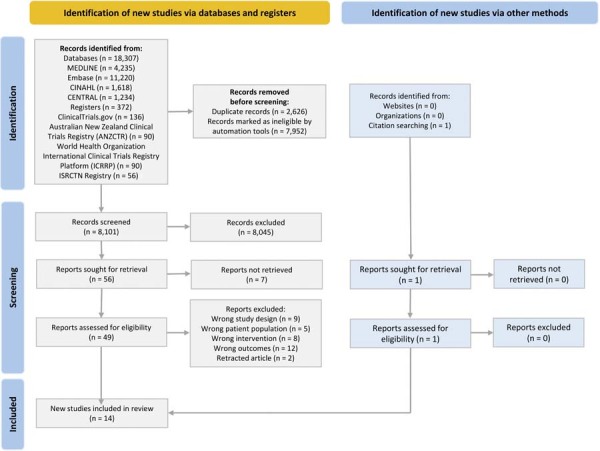
Preferred Reporting Items for Systematic Reviews and Meta-Analyses (PRISMA) flow diagram of search strategy and included studies.

All Bayesian analyses were performed using a minimally informative prior: a normal distribution for the log-RR with a mean of zero (centred on no effect) and assuming a 95% probability that the effect lies between RR values of 0.5 and 2 (SD of 0.355). A weakly informative half-normal prior distribution was defined for the heterogeneity parameter. Minimally informative and weakly informative priors were selected based on previous recommendations in the literature regarding predictive distributions in Bayesian analysis.^(29,30)^In sensitivity analyses, the treatment effect prior probability distribution was defined by setting an optimistic, pessimistic, and minimally informative prior belief for the treatment effect.^(31)^The pessimistic and optimistic priors were informed by the range of effect size estimates from previous studies, suggesting the effect of albumin ranging from a reduction of 10% in the risk of AKI to an increase of 3%^(32,33)^(OR = 0.90 for the optimistic prior and OR = 1.03 for the pessimistic prior). The optimistic prior SD was defined to retain a 0.15 probability of harm [Pr(OR > 1)] (SD = 0.10), and the pessimistic prior was defined to retain a 0.30 probability of harm [Pr(OR < 1)] (SD = 0.06). Further details on the selection of priors are described in the study protocol, and the density distributions of the different priors are shown in figure 1S (Supplementary Material).

The PRISMA flow diagram was designed using the PRISMA 2020 R package and Shiny app.^(48)^

Quantitative heterogeneity was assessed using posterior estimates of the heterogeneity parameter (tau) and its 95%CrI. The proportion of variation across studies owing to heterogeneity rather than chance was assessed with the I^2^ statistic.

The following prespecified subgroup analyses were performed: albumin concentration (isooncotic 4 - 5% *versus* hyperoncotic 20 - 25%); timing of therapy (intraoperative only *versus* intra and/or postoperative); type of procedure (isolated only *versus* isolated or combined); risk of bias (low *versus* high or unclear), and type of comparator fluid (crystalloid *versus* colloid or both). Subgroup heterogeneity was assessed by including an interaction term in the Bayesian analysis to obtain an estimate and a 95%CrI for the ratio of RRs (RRRs) from the posterior distribution of the interaction estimate. The presence of small-study effects was assessed visually using funnel plots and formally with Egger’s regression test.

All statistical analyses were performed with R version 4.3.3 using Bayesmeta and meta packages.^(34,35)^

### Confidence in the cumulative evidence

The Grading of Recommendations, Assessment, Development and Evaluation (GRADE) criteria^(36)^were independently applied in duplicate to assess the certainty of the evidence regarding key outcomes as prespecified in the protocol.

## RESULTS

From 18,680 records, 14 eligible randomized clinical trials, including 3,304 adult participants who had undergone on-bypass cardiac surgery, were included (731 female [22%]).^(21,22,33,37-47)^ The search results and reasons for exclusion are detailed in figure 1 and table 1S (Supplementary Material).

Table 1 presents the characteristics of included trials. Further details of fluid regimens, trial characteristics, and outcome definitions are presented in tables 2S - 6S (Supplementary Material). All trials were published in peer-reviewed journals, except one, for which no information on peer review was available.^(41)^Aggregate-level data from one unpublished trial, as well as additional unpublished aggregate-level data from five trials, were obtained directly from trial authors (Table 7S - Supplementary Material).^(21,22,42,44,45,47)^

**Table 1:** Characteristics of included randomized clinical trials

Source	Setting	Participants* n (% female)		Age†, year		Study fluid		Study fluid timing‡; Indication§	Procedure type* n (%)		Outcomes#
		Albumin	Comparator	Albumin	Comparator	Albumin	Comparator		Isolated procedure¶	Combined procedure ||	
Wigmore et al.^(^^21^^)^	Multiple centre, Australia	233 (26.6)	233 (26.6)	65 (12)	66 (11)	Albumin 20%	Crystalloid	Postoperative; volume replacement	383 (82.2)	83 (17.8)	AKI Mortality ICU LOS Hospital LOS Duration of ventilation Duration of vasopressor RRT
Shehabi et al.^(^^22^^)^	Multiple centre, Australia, Italy	307 (43.5)	304 (33.9)	69.12 (11.02)	68.88 (10.55)	Albumin 20%	Standard care**	Postoperative; continuous infusion	229 (37.5)	382 (62.5)	AKI Mortality ICU LOS Hospital LOS Duration of ventilation Duration of vasopressors RRT
Skhirtladze et al.^(^^33^^)^	Single centre, Austria	76 (30.3)	81 (35.8) 79 (22.8) ††	66 (23/85)	67 (28/87) 67 (24/87) ††	Albumin 5%	HES 6%, Ringer’s lactate††	CPB prime, intraoperative, postoperative; volume replacement	136 (57.6)	100 (42.4)	Mortality ICU LOS Hospital LOS Duration of ventilation RRT
Adriane et al,^(^^37^^)^	Single centre, Indonesia	40 (12.5)	40 (17.5) 40 (12.5) ‡‡	NR	NR	Albumin 4%	Gelatine Ringer’s lactate ‡‡	Postoperative; continuous infusion	120 (100)	0 (0)	ICU LOS Hospital LOS Duration of ventilation
Diehl et al.^(^^38^^)^	Single centre, USA	27 (25.9)	33 (12.1)	56.6 (8.1)	58.0 (8.0)	Albumin 5%	HES 6%	Postoperative; continuous infusion	60 (100)	0 (0)	Mortality ICU LOS Hospital LOS
Duncan et al.^(^^39^^)^	Single centre, USA	72 (38.9)	69 (31.9)	69 (9)	71 (10)	Albumin 5%	HES 6%	Intraoperative; volume replacement	67 (47.5)	74 (52.5)	AKI Mortality RRT
Fitzgerald et al.^(^^40^^)^	Single centre, USA	17 (17.6)	13 (15.4)	59.6 (12.1)	65.6 (6.1)	Albumin 25% Albumin 5%	Standard care §§	CPB prime, intraoperative, postoperative; volume replacement	30 (100)	0 (0)	AKI Mortality ICU LOS Duration of ventilation
George et al.^(^^41^^)^	Single centre, France	15 (NR)	15 (NR)	56 (4)	62 (7)	Albumin 4%	Dextran 40	Postoperative; continuous infusion, volume replacement	30 (100)	0 (0)	AKI Mortality
Kirklin et al.^(^^42^^)^	Single centre, USA	15 (NR)	15 (NR)	NR	NR	Albumin 5%	HES 6%	Intraoperative, postoperative; continuous infusion	30 (100)	0 (0)	Mortality ICU LOS Duration of ventilation RRT
London et al.^(^^43^^)^	Single centre, USA	44 (0)	50 (0)	64 (7)	63 (7)	Albumin 5%	Pentastarch 10%	Postoperative; volume replacement	86 (91.5)	8 (8.5)	Mortality ICU LOS Hospital LOS Duration of ventilation RRT
Munsch et al.^(^^44^^)^	Single centre, United Kingdom	20 (15)	20 (15)	55 (42/70)	59 (43/73)	Plasma protein fraction ¶¶	HES 6%	Postoperative; volume replacement	40 (100)	0 (0)	AKI Mortality Duration of ventilation
Pesonen et al.^(^^45^^)^	Single centre, Finland	693 (20.8)	693 (21.8)	65 (10)	65 (10)	Albumin 4%	Ringer’s acetate	CPB Prime, intraoperative, postoperative; volume replacement	997 (71.9)	389 (28.1)	AKI Mortality ICU LOS RRT
Tølløfsrud et al.^(^^46^^)^	Single centre, Norway	10 (30)	10 (20) 10 (20) 10 (20)||||	62 (9)	65 (4) 68 (5) 63 (5)||||	Albumin 4%	Ringer’s acetate Polygeline Dextran 70||||	Intraoperative, postoperative; volume replacement	40 (100)	0 (0)	Mortality Duration of ventilation
Wahba et al.^(^^47^^)^	Single centre, Germany	10 (10)	10 (20)	63 (7)	66 (7)	Albumin 5%	Polygeline	Postoperative; continuous infusion, volume replacement	20 (100)	0 (0)	Duration of ventilation

AKI - acute kidney injury; ICU - intensive care unit; LOS - length of stay; RRT - renal replacement therapy; NR - not reported; HES - hydroxyethyl starch; CPB - cardiopulmonary bypass.

* Participants and procedure types are presented as counts (percentage); † age is presented as mean (SD) or median (IQR 25th/75th percentile). All included trials recruited adult participants only. No trials included in the review had paediatric populations; ‡ study fluid timing considered as the time point at which the fluid was given, classified as one or more of; cardiopulmonary bypass priming, intraoperative fluid therapy, or postoperative fluid therapy; § study fluid indication considered as the indication for fluid therapy, classified as one or more of; continuous infusion or volume replacement; ¶ isolated procedures include any of the following; isolated coronary artery bypass surgery, isolated single valve procedures, isolated procedures involving the ascending aorta or aortic root. Procedure type as reported in included trials are detailed in table 3S (Supplementary Material); || combined procedures include procedures involving two or more of: coronary artery bypass grafting, valve procedures, procedures involving the ascending aorta or aortic root. Procedure type as reported in included trials are detailed in table 3S (Supplementary Material); # outcomes as reported in included trials: definitions for each outcome as described by trial authors are detailed in tables 5S - 6S (Supplementary Material). Definitions of kidney injury have been unified using the Kidney Injury Improving Global Outcomes (KDIGO) criteria as previously specified in the published protocol. Duration of ventilation refers to duration of mechanical ventilation. Duration of vasopressors refers to duration of vasopressor or inotrope therapy postoperatively; ** standard care defined as any routine treatment at each institution. No 20% albumin for the first 24 hours in the standard care arm, 20% albumin accepted after 24 hours. Any other fluids or blood products accepted including 4-5% albumin throughout study period;^(^^22^^)^

†† listed numbers 1) and 2) in participant, age, and study fluid columns correlate with listed comparator study fluids as follows 1) HES 6%, 2) Ringer’s Lactate; ‡‡ listed numbers 1) and 2) in participant, age, and study fluid columns correlate with listed comparator study fluids as follows 1) Gelatine, 2) Ringer’s Lactate; §§ standard care defined as control group. Fluid selection and administration selected at the point of care according to institutional protocols;^(^^40^^)^ ¶¶ plasma protein fraction solution deemed equivalent to 4% albumin as per definition by Winkler et al.;^(^^49^^)^ plasma protein fraction is prepared as a 5% solution, is derived from human plasma, and contains at least 83% albumin; |||| listed numbers 1) to 3) in participant, age, and study fluid columns correlate with listed comparator study fluids as follows 1) Ringer’s Acetate, 2) Polygeline, 3) Dextran 70.

The 14 included trials had a median of 77 participants (interquartile range [IQR], 30 - 236). The mean age of trial participants was 65.27 years (SD = 16.46 years). The mean European System for Cardiac Operative Risk Evaluation (EuroSCORE) for the five trials with available data was 2.87 (SD = 3.41).^(21,22,33,40,45)^

Albumin solution was studied in 4 - 5% preparations in ten trials,^(33,37-39,41-43,45-47)^ 20% preparation in two trials,^(21,22)^while one trial included 5% and 25% preparations.^(40)^ A clinical trial by Munsch et al.^(44)^studied plasma protein fraction, considered equivalent to 4% albumin for analysis based on albumin concentration.^(49)^

### Risk of bias

**Figure 2:**
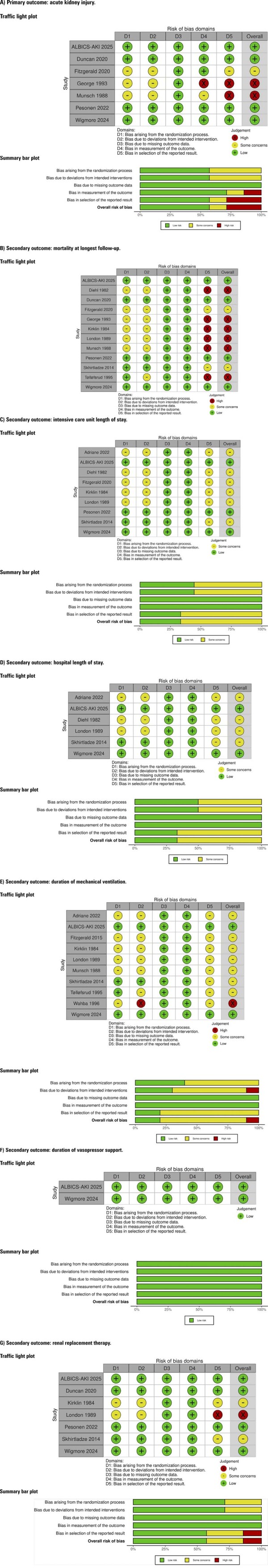
Risk of bias assessments for primary and secondary outcomes.

Risk of bias assessments are presented in figure 2. Of the 14 included trials, four were assessed as having an overall low risk of bias across all domains and outcomes.^(21,22,39,45)^ The overall risk of bias was adjudicated as low for four of the seven trials, including 2,590 of the 2,690 participants (96.3%) contributing data to the primary outcome of acute kidney injury.^(21,22,39,45)^

Traffic light plots and summary bar plots were designed using the Risk-of-bias VISualization (robvis) R package and Shiny web app.^(^^50^^)^

### Primary outcome

There were seven randomized clinical trials, including 2,690 participants, which contributed data to the primary outcome. The definitions used for AKI by trial authors differed; three trials (n = 2,449) used the KDIGO criteria,^(5,21,22,45)^ one trial (n = 141) used the Risk, Injury, Failure, Loss, End Stage (RIFLE) criteria,^(5,39)^ one trial (n = 30) used the Society of Thoracic Surgeons criteria,^(40,51)^ two trials (n = 70) did not provide a definition of renal dysfunction^(41,44)^ (Table 4S - Supplementary Material).

**Figure 3:**
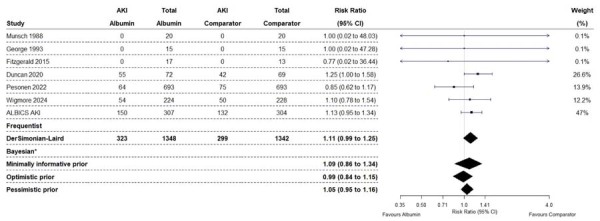
Comparison between albumin *versus* comparator fluids for acute kidney injury.

**Figure 4:**
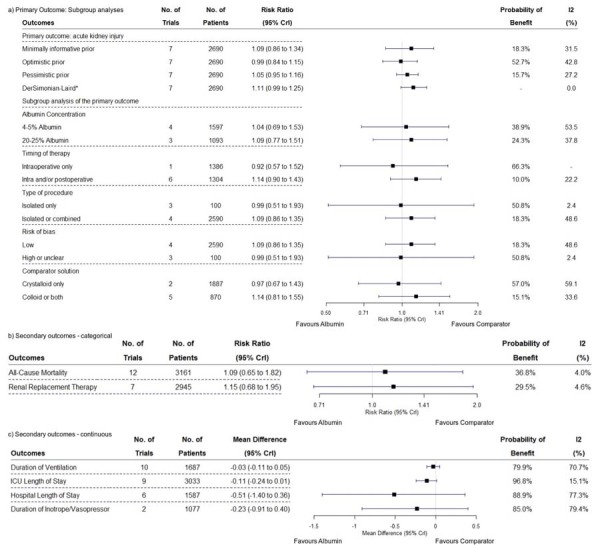
Primary outcome, secondary outcomes, and subgroup analyses for the comparison between albumin *versus* comparator fluids.

**Figure 5:**
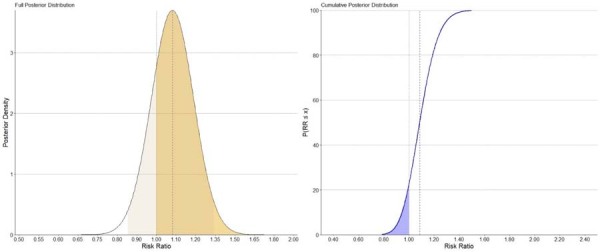
Posterior probability of the risk ratio for acute kidney injury for albumin compared with comparator fluids.

Using a Bayesian random effects model with minimally informative priors, the pooled estimated RR for AKI for albumin solutions compared with comparator fluids was 1.09 (95%CrI 0.86 to 1.34, τ = 0.12; I^2^= 31.5%) with an 18.3% posterior probability that albumin solutions were associated with reduced AKI (Figures 3 - 5; Table 8S [Supplementary Material]). The certainty of evidence was adjudicated as high, as presented in table 2. The results of sensitivity analyses using semi-informative priors, studies with low risk of bias, and frequentist methods were consistent with the primary analysis (Figure 4; Table 8S [Supplementary Material]). There was no evidence of small-study effects by visual assessment of the contour-enhanced funnel plots or by regression-based Egger’s test (Figure 2SA - Supplementary Material).

*Credible intervals are presented for Bayesian analysis. The blue boxes represent point estimates, and the whiskers represent 95% confidence intervals. The displayed box weights are based on Bayesian analysis with minimally informative priors. The overall pooled estimate is depicted by diamonds, with the width representing the credible interval for Bayesian analysis and the confidence interval for frequentist analysis, and the middle representing the point estimate. Different priors are shown for Bayesian analysis. AKI - acute kidney injury; 95%CI - 95% of confidence interval.

**Table 2:** Grading of Recommendations Assessment, Development and Evaluation (GRADE) Summary of Findings

Outcome	Number. of trials / number. of participants	Certainty of evidence*	Study fluid n/n (%) or mean†		Absolute difference (95%CrI)	Risk ratio (95%CrI)
			Albumin	Comparators		
Acute kidney injury	7/2,690	⨁⨁⨁⨁ High	323/1,348 (24.0%)	299/1,342 (22.3%)	0.02 (-0.031 to 0.076)	1.09 (0.86 to 1.34)
Mortality	12/3,161	⨁⨁⨁◯ Moderate‡	15/1,528 (1.0%)	13/1,633 (0.8%)	0.001 (-0.003 to 0.007)	1.09 (0.65 to 1.82)
ICU length of stay (days)	9/3,033	⨁⨁⨁◯ Moderate§	2.3	3.0	-0.11 (-0.24 to 0.01)	NA
Hospital length of stay (days)	6/1,587	⨁⨁◯◯ Low¶	12.1	14.9	-0.51 (-1.40 to 0.36)	NA
Duration of mechanical ventilation (hours)	10/1,687	⨁⨁⨁◯ Moderate||	0.57	0.64	-0.03 (-0.11 to 0.05)	NA
Duration of vasopressor support (days)	2/1,077	⨁⨁◯◯ Low¶	1.4	1.7	-0.23 (-0.91 to 0.40)	NA
Renal replacement therapy	7/2,945	⨁⨁⨁◯ Moderate‡	16/1,430 (1.1%)	12/1,515 (0.8%)	0.001 (-0.003 to 0.008)	1.15 (0.68 to 1.95)

95%CrI - 95% credible interval; ICU - intensive care unit; NA - not applicable. * The certainty of the evidence is based on the GRADE approach.^(^^36^^)^ This is a system designed to rate the quality of a body of evidence. This approach creates four levels of evidence certainty: high, moderate, low and very low. High certainty (⨁⨁⨁⨁) – very confident that the true effect lies closest to that of the estimate of effect; moderate certainty (⨁⨁⨁◯) – the true effect is likely to be close to the estimate of effect, but there is the possibility that it is substantially different; low certainty (⨁⨁◯◯) – confidence in the effect estimate is limited and the true effect may be substantially different from the estimate; very low certainty (⨁◯◯◯) – very little confidence in the effect estimate and the true effect is likely to be substantially different from the estimate;^(^^36^^)^ † acute kidney injury, mortality, and renal replacement therapy are presented as counts (percentage); intensive care unit length of stay, hospital length of stay, duration of mechanical ventilation and duration of vasopressor therapy are presented as pooled means; ‡ downgraded due to imprecision (absolute effect fails to include or exclude clinically important benefit or harm with low absolute event rate); § downgraded due to risk of bias (large number of patients (as a weighted proportion) are from studies with unclear risk of bias); ¶ downgraded due inconsistency (study heterogeneity) and imprecision (absolute effect fails to include or exclude clinically important benefit or harm with low absolute event rate); || downgraded due to inconsistency (study heterogeneity).

*Confidence intervals are presented for frequentist analysis. Figure A shows the primary outcome with both sensitivity and subgroup analyses. Figure B shows categorical secondary outcomes, and figure C shows continuous secondary outcomes. The blue boxes represent point estimates, and the whiskers represent credible intervals for Bayesian analyses and confidence intervals for frequentist analyses. Different priors are shown for Bayesian analysis. 95%CrI - 95% credible interval; ICU - intensive care unit.

Left graph, the full posterior distribution of the estimated risk ratio, with the dashed vertical line indicating the median value and the area highlighted in tan indicating the percentile-based 95% credible interval. The orange area corresponds to a risk ratio greater than 1 (i.e., the intervention is associated with a higher incidence of acute kidney injury than comparator fluids). The dotted line at a risk ratio of 1 indicates no treatment effect. The figure demonstrates that the probability that albumin is associated with a reduced risk of acute kidney injury (to any extent) compared with comparator fluids is 18.3%. Right graph, the cumulative posterior distribution of the estimated risk ratio, with the y-axis corresponding to the probability that the RR is less than or equal to the value on the x-axis. The blue-grey area indicates a beneficial intervention (i.e., risk ratio lower than 1). The dashed vertical line indicates the median value, and the dotted line at a risk ratio of 1 indicates no treatment effect. RR - risk ratio.

### Subgroup analyses

The primary outcome of AKI was assessed in the prespecified subgroups (Figure 4). No data were available for the prespecified subgroup with preoperative estimated glomerular filtration rate (eGFR) less than 60mL/min/1.73m^2^, so this was not included. As presented in figure 4 and table 8S (Supplementary Material), there were no statistically significant subgroup effects.

### Secondary outcomes

Secondary outcomes are presented in table 2, figure 4, and table 8S and figures 3S - 8S (Supplementary Material). Assessment of small-study effects is presented in figure 2SB - 2SG (Supplementary Material). Among the 12 studies contributing data on mortality, 15/1,528 (0.98%) participants in the albumin group and 13/1,633 (0.80%) participants in the comparator group died. Bayesian meta-analysis demonstrated the use of albumin solutions was associated with no significant difference in mortality (RR 1.09; 95%CrI 0.65 - 1.82, moderate certainty) and no statistically significant subgroup effects (Figure 3SA - 3SC - Supplementary Material). Compared with the use of comparator fluids, Bayesian meta-analysis demonstrated the use of albumin solutions was associated with no significant difference in ICU length of stay (MD -0.11 days; 95%CrI -0.24 - 0.01, moderate certainty) (Figure 4SA - 4SB - Supplementary Material), hospital length of stay (MD -0.51 days; 95%CrI -1.40 - 0.36, low certainty) (Figure 5SA - 5SB - Supplementary Material), duration of mechanical ventilation (MD -0.03 hours; 95%CrI -0.11 - 0.05, moderate certainty) (Figure 6SA - 6SB - Supplementary Material), duration of vasopressor support (MD -0.23 days; 95%CrI -0.91 - 0.40, low certainty) (Figure 7SA - 7SB - Supplementary Material), or RRT (RR 1.15; 95%CrI 0.68 - 1.95, moderate certainty) (Figure 8SA - 8SB - Supplementary Material). Four secondary outcomes with certainty of evidence assessed as moderate were downgraded due to imprecision (mortality and RRT), a large proportion of data from studies with high risk of bias (ICU length of stay), or heterogeneity (duration of mechanical ventilation). Two secondary outcomes with certainty of evidence assessed as low (hospital length of stay and duration of vasopressor support) were both downgraded due to heterogeneity and imprecision.

## DISCUSSION

This systematic review of 14 randomized controlled trials incorporating 3,304 patients demonstrated that, compared with other fluid regimens, albumin fluid therapy does not reduce the risk of CSA-AKI. The Bayesian analysis found no significant difference in the primary outcome with albumin solutions compared with comparator regimens, with the point estimate suggesting a 9% relative increase in the risk of CSA-AKI and low probability (18.3%) that albumin-containing fluids reduce the risk of AKI, with a high level of certainty for these findings. These findings were consistent with the sensitivity analysis and the secondary frequentist analysis, which supported a null hypothesis. The Bayesian and frequentist analyses also found no benefits for mortality, length of stay, RRT, duration of vasopressors, or duration of mechanical ventilation. However, the strength of these findings is less certain.

This analysis builds on evidence from previous meta-analyses and clinical guidelines evaluating the use of albumin fluid in cardiac surgery. The inclusion of additional results from two recent large randomized trials addressed limitations of previous reviews and enabled analysis of hyperoncotic albumin therapy that had not previously been possible.^(19-22,52)^ The findings are consistent with previous meta-analyses in surgical and other critically ill populations.^(19,53,54)^ They are also consistent with the literature evaluating the use of albumin in cardiopulmonary bypass priming, which has found no significant benefit with albumin priming strategies.^(55)^ Other studies suggest outcomes may depend on context, for instance, in patients with hypoalbuminaemia. Observational studies have described a potential association between cardiac surgical patients with hypoalbuminaemia in the preoperative and postoperative settings and poor clinical outcomes, including AKI.^(56,57)^In this context, one randomized trial found that preoperative hyperoncotic albumin administration in off-bypass cardiac surgery for patients with preoperative hypoalbuminaemia was associated with reduced postoperative AKI.^(32)^ A consideration when comparing trials evaluating off-bypass cardiac surgery with the current review is that the administration of albumin may not completely ameliorate the complex physiological insult following cardiopulmonary bypass, which includes microcirculatory dysfunction and endothelial disruption.^(58,59)^ While the pathophysiological mechanisms of albumin in this context are poorly understood, and some benefits of albumin have been postulated, there is some conflicting evidence.^(14,16)^For instance, one animal study in the cardiac arrest population reported worsening AKI with exogenous albumin, attributed to tubular apoptosis and increased relative glomerular filtration.^(60)^Previous reviews and meta-analyses have considered both off-bypass and on-pump cardiac surgery.^(19,20,23)^An important distinction of this review is the intentional exclusion of off-bypass trials, due to the unique risk factors associated with cardiopulmonary bypass.

This study has several strengths. Previous meta-analyses and the ICTMG guidelines acknowledged insufficient evidence to make a strong recommendation against the use of albumin in major surgery due to small trials and heterogeneous study designs.^(18-20)^This analysis includes over 1,000 additional participants and demonstrates low heterogeneity, thereby increasing the precision and certainty of estimates of the effect of albumin-containing infusions on clinical outcomes. With the inclusion of the recent trials, over 90% of the data for the primary outcome are from studies with low risk of bias, and outcomes for the use of hyperoncotic albumin solutions are available, in addition to the isooncotic solutions assessed in previous studies. Finally, the use of a Bayesian analysis increases the robustness of the analysis. It indicates that the probability of subsequently revealing a beneficial effect of albumin-containing fluids in this context is low.

This study has several limitations. First, the definition and timing of AKI outcomes varied across trials. Second, the intervention and comparator fluid regimen varied between trials, potentially contributing to heterogeneity. To address this limitation, a network meta-analysis would be an alternative methodological approach for future studies to provide nuanced insight into the impact of different fluid regimens on clinical outcomes. Nonetheless, the pair-wise meta-analysis performed in this study was chosen based on a hypothesis that the concentration of albumin or comparator fluid regimens was unlikely to be a significant modifier of the treatment effect, as demonstrated in recent related pair-wise and network meta-analyses.^(19,61)^ Analyses of heterogeneity and subgroup analyses of albumin concentration and comparator solution in this meta-analysis were consistent with this hypothesis. Third, some regimens included the potential administration of albumin-containing fluid in the comparator regimen; these trials demonstrated group separation in serum albumin concentration, and a subgroup analysis of crystalloid comparators was consistent with the primary analysis.^(21,22)^Fourth, individual patient and patient subgroup data were not available to assess whether certain patient subgroups or perioperative factors are associated with benefit or, conversely, increased risk of AKI from this intervention. For instance, there was no subgroup data on procedure type for any trials that studied multiple procedures, despite these characteristics potentially impacting clinical outcomes. Nonetheless, baseline characteristics of included studies, including preoperative renal function, risk scores, and bypass duration, were balanced between groups, and there were no significant subgroup effects in the subgroup analysis of type of procedure by trial (trials investigating isolated procedures only *versus* trials investigating isolated and combined procedures) in this meta-analysis.^(21,22,33,37-47)^ Furthermore, a subgroup analysis in one large included trial found no significant differences in outcomes among high-risk subgroups, particularly participants with reduced preoperative eGFR and prolonged cardiopulmonary bypass duration.^(22)^ To gain greater insight into potentially important patient and perioperative factors, an individual patient data meta-analysis would be an informative avenue for future research. Fifth, a minimal clinically important difference (MCID) calculation was not performed in the current analysis. This was determined based on the analysis results, which demonstrate that the probability of any benefit from albumin fluid therapy in this context is low, as well as the cost implications of albumin fluids and patient preferences regarding blood products.^(62,63)^ It was deemed that an MCID calculation was likely not indicated in this instance, as the current findings are sufficient to justify the conclusions. Finally, the certainty of evidence regarding mortality, RRT, mechanical ventilation, vasopressor support, and length of stay was moderate or low due to the quality of the evidence and low event rates.

This analysis strengthens the evidence base and provides a rationale for reviewing the use of albumin-containing fluids in the perioperative care of cardiac surgical patients, particularly given patients’ preferences to avoid blood products and the increased cost of albumin.^(62,63)^ Albumin solutions are considerably more expensive than other available solutions used in fluid therapy and resuscitation. According to economic data from 187 intensive care units across 22 countries, published in the FLUID TRIPS study,^(62)^ the average cost of isooncotic and hyperoncotic albumin fluids per millilitre was 4 - 27 times higher than that of other crystalloid or colloid solutions.^(62)^ Retrospective studies by Fink et al.^(64)^ and Rabin et al.^(65)^ found restrictive albumin prescribing practices were associated with substantial financial benefits, describing cost savings in each single-centre cardiac ICU between USD $10,000 and $45,000 per month.^(64,65)^Given these economic implications and considering the significant impact of CSA-AKI, there remains a need to identify alternative interventions to reduce the risk of this important complication.

This study did not suggest any significant subgroup interaction with respect to the timing or concentration of albumin fluids, suggesting that these results are generalisable to any albumin solution. Thus, ongoing research efforts should consider prioritizing further investigation of other interventions that have shown potential benefits in initial randomized trials, such as amino-acid infusion and extracorporeal blood purification.^(66,67)^

## CONCLUSION

Among patients undergoing on-bypass cardiac surgery, it is unlikely that the use of albumin-containing fluid reduces the risk of cardiac surgery-associated acute kidney injury. Other interventions are needed to reduce the risk of this condition.
